# Transcutaneous electrical nerve stimulation for advanced cancer pain inpatients in specialist palliative care—a blinded, randomized, sham-controlled pilot cross-over trial

**DOI:** 10.1007/s00520-020-05370-8

**Published:** 2020-03-03

**Authors:** Waldemar Siemens, Christopher Boehlke, Michael I. Bennett, Klaus Offner, Gerhild Becker, Jan Gaertner

**Affiliations:** 1 Clinic for Palliative Care, Medical Center, Faculty of Medicine, University of Freiburg, Robert-Koch-Str 3, 79106 Freiburg, Germany; 2 Academic Unit of Palliative Care, Leeds Institute of Health Sciences (LIHS), School of Medicine, University of Leeds, Leeds, UK; 3 Department of Anesthesiology and Critical Care, Medical Center, Faculty of Medicine, University of Freiburg, Freiburg, Germany; 4 Center for Palliative Care Hildegard, Basel, Switzerland

**Keywords:** Palliative care, Cancer pain, Non-pharmacological, Transcutaneous electrical nerve stimulation, Complementary therapies

## Abstract

**Purpose:**

Transcutaneous electrical nerve stimulation (TENS) is a treatment option for cancer pain, but the evidence is inconclusive. We aimed to evaluate the efficacy and safety of TENS.

**Methods:**

A blinded, randomized, sham-controlled pilot cross-over trial (NCT02655289) was conducted on an inpatient specialist palliative care ward. We included adult inpatients with cancer pain ≥ 3 on an 11-point numerical rating scale (NRS). Intensity-modulated high TENS (IMT) was compared with placebo TENS (PBT). Patients used both modes according to their preferred application scheme during 24 h with a 24-h washout phase. The primary outcome was change in average pain intensity on the NRS during the preceding 24 h. Responders were patients with at least a “slight improvement.”

**Results:**

Of 632 patients screened, 25 were randomized (sequence IMT-PBT = 13 and PBT-IMT = 12). Finally, 11 patients in IMT-PBT and 9 in PBT-IMT completed the study (*N* = 20). The primary outcome did not differ between groups (IMT minus PBT: − 0.2, 95% confidence interval − 0.9 to 0.6). However, responder rates were higher in IMT (17/20 [85%] vs. 10/20 [50%], *p* = 0.0428). Two patients experienced an uncomfortable feeling caused by the current, one after IMT and one after PBT. Seven patients (35%) desired a TENS prescription. Women and patients with incident pain were most likely to benefit from TENS.

**Conclusion:**

TENS was safe, but IMT was unlikely to offer more analgesic effects than PBT. Even though many patients desired a TENS prescription, 50% still reported at least “slight pain relief” from PBT. Differences for gender and incident pain aspects demand future trials.

**Electronic supplementary material:**

The online version of this article (10.1007/s00520-020-05370-8) contains supplementary material, which is available to authorized users.

## Introduction

Cancer pain is a leading symptom on palliative care units (ca. 80% of patients) [1, 2]. As an adjunct to pharmacological cancer pain management, transcutaneous electrical nerve stimulation (TENS) is a safe, non-invasive, and inexpensive non-pharmacological option for pain treatment [3–5]. TENS is usually applied at the site of pain, where it stimulates large diameter (A-β) afferent fibers, which leads to a decreased activity of transmission cells and subsequently to reduced perception of pain according to the Gate-Control-Theory [6].

Though TENS is recommended in most palliative care and cancer pain textbooks [3, 4], controlled trials from a palliative care setting are lacking, what may be due to multimorbidity, recruitment barriers, high attrition rates, and general ethical difficulties [7]. In a Cochrane Review on TENS for cancer pain in adult patients, only three RCTs were identified [8]. Varying study designs, TENS modes, and outcome measures across studies as well as small sample sizes led to inconclusive results [9–11].

Therefore, the primary aim was to evaluate efficacy and safety of TENS in addition to standard care for advanced cancer pain patients. The secondary aim of this study was the exploratory identification of subgroups that do or do not benefit from TENS.

## Materials and methods

### Study design and setting

This was a blinded, randomized, sham-controlled pilot cross-over trial (DRKS00007990; ClinicalTrials.gov Identifier NCT02655289). Patients were recruited from the inpatient specialist palliative care ward and the acute pain service of the University Medical Center Freiburg, Germany.

The study was approved by the local ethics committee in 2015. We report this manuscript in accordance with the Consolidated Standards of Reporting Trials (CONSORT) Statement’s extension for non-pharmacological treatments (NPTs) (Online Resource 1) [12] and the Template for Intervention Description and Replication (TIDieR) checklist (Online Resource 2) [13].

### Participants

We included adult inpatients ≥ 18 years with cancer and pain ≥ 3 on an 11-point numerical rating scale (NRS, 0 = no pain; 10 = pain as bad as you can imagine) in the preceding 24 h. Pain could have been caused by the tumor itself (cancer pain), cancer-directed therapy, or by an association with the tumor, e.g., being bedridden or daily activities. Patients had to have received specialist palliative care for at least 24 h in the inpatient palliative care ward or by the acute pain service. There were no limitations concerning tumor site and type of cancer pain (neuropathic and nociceptive pain).

The exclusion criteria were verbal or cognitive inability to use TENS or to answer the questionnaire, high probability of dying within the next week according to the treating physician, and pain that was not directly or indirectly related to the tumor (i.e., chronic low back pain). In addition, we used the following TENS contraindications [4, 14]: electronic implants like pacemakers, metal implant on electrode site, arrhythmia, pregnancy, epilepsy, dermatological conditions or frail skin on electrode site, and history of allergy regarding electrodes or patches.

### Intervention: intensity-modulated high TENS

A dual channel TENS device (ARTROSTIM® SELECT™, ORMED) was used at the site of pain in the intervention (IMT) and placebo TENS (PBT) phase. Patients were instructed by an experienced researcher who received training and supervision of a senior physician. The intervention used *intensity*-*modulated* TENS (IMT) with 100 Hz. The patients were advised to choose a “strong but comfortable” intensity [5, 15] and the intensity was modulated automatically with a decrease of 40% every 0.5 s in order to prevent habituation [16].

IMT and PBT were used by patients individually, i.e., they were free to turn TENS on or off as they pleased, according to their own perceived benefit.

### Comparison: Placebo TENS

PBT, or sham-controlled TENS, was based on the *continuous* mode with 100 Hz and a fixed intensity, which was either slightly or not perceptible at all. For the sake of adequate blinding, we instructed the patients that two active TENS modes were compared. Patients were informed that the TENS device could be turned on according to individual needs and that the sensory threshold would probably not be reached in this TENS mode. The TENS device was activated for PBT with an intensity that was mostly perceptible for a few seconds and then fell below the sensory threshold through habituation. The display and flashing light of the device in the PBT mode looked and behaved exactly as in the IMT mode [17].

### Outcomes

Most outcomes were based on the Brief Pain Inventory (BPI) [18, 19] and were chosen with consideration to recommendations for pain assessment [17, 20].

The primary outcome was change of mean pain intensity in the preceding 24 h, measured on an 11-point NRS before and after the 24-h IMT or PBT phase and after the flexible follow-up.

Secondary outcomes of the BPI over the 24 h period included change of worst pain intensity, change of least pain intensity, and BPI items that may have been affected by pain: general activity, mood, walking ability, normal work, relations with other people, sleep, and enjoyment of life. Some outcomes were measured on other scales: change of pain perception during TENS application on a 7-point verbal rating scale (VRS), number and percentage of responders defined as patients with at least a “slight improvement” on the abovementioned 7-point VRS. Question 30 from the European Organization for Research and Treatment of Cancer quality of life questionnaire C30 (EORTC QLQ-C30) [21] was used to assess quality of life.

Medication with an influence on pain (opioids, non-opioids, antidepressants, anticonvulsants) was documented at baseline and during the study, i.e., if a new drug was added or removed, or if the dose of a drug was increased or decreased by > 50% [22]. Furthermore, the oral morphine equivalent dose (MED) was calculated. Pain classification was assessed with the Edmonton Classification System for Cancer Pain [23] and the Douleur Neuropathique en 4 Questions (DN4) [24].

### Study procedure

The patients were screened by treating physicians in the inpatient palliative care ward and the acute pain service. Eligible patients were contacted by the study team to obtain informed consent for study participation. A senior physician was responsible for the randomization list, enabled a concealed central allocation, and patients were directly randomized after giving informed consent. Randomization was performed according to a random, computer-generated list with an allocation ratio of 1:1.

The senior physician was not involved in the provision of the intervention or data assessment. TENS was started immediately after allocation to the sequence, and patients were instructed that two active TENS modes were compared in this study.

Patients, researchers, the outcome assessor, and the biometrician were blinded concerning the mode of intervention. Thus, the researcher placed the electrodes without knowing the TENS mode. TENS was subsequently activated (IMT or PBT) by a non-blinded treating physician who was not involved in the data collection, data analysis, or the preparation of the manuscript. Patients were instructed not to tell anything about their perceptions of the TENS mode to the research team and had to turn off the TENS device during answering the questionnaire.

Patients used the first TENS mode during the first 24-h phase (period 1). After the 24 h washout phase, the patient crossed over to the other TENS mode for another 24 h (period 2). After the main part of the study (period 1 and period 2), patients could decide whether to continue with one of the TENS modes (IMT or PBT) for a flexible short-term follow-up or to stop the study (see study design: Online Resource 3).

### Statistical analysis

We aimed to include 20 patients in this pilot trial to assess the effects and safety (primary aim), and gather valuable information for a fully powered multicenter study. This cross-over trial was analyzed according the recommendations by Wellek and Blettner [25] and Li et al. [26]. Unpaired *t* tests of the within-subject sums of the result from both periods were used to check carry-over effects (*p* ≥ 0.05: carry-over effect is negligible) [25]. Paired *t* tests were calculated for within-subject differences of change scores from both periods (t2 minus t1, or t4 minus t3, see Online Resource 3). As sensitivity analysis, paired *t* tests were also calculated for within-subject differences of post treatment scores from both periods (t2, t4), according to the recommendations by Li et al. [26]. Numbers of responders were compared with the help of the chi-squared test.

As subgroup analysis (secondary aim), we explored characteristics from patients that benefited or did not benefit from TENS regarding average pain intensity in the preceding 24 h. Benefit was defined as difference of more than minus one for change scores (primary outcome) and post treatment scores when subtracting IMT minus PBT, and/or change scores within groups (IMT, PBT) of more than minus one on the NRS [27].

Patients with complete data for period 1 and period 2 were analyzed per-protocol [28] and were evaluated irrespective of how often or how long they used the TENS modes.

All tests for the secondary outcomes were exploratory. The tests were performed two-tailed using an alpha level of 0.05. The statistical analysis was performed using R (RStudio Version 3.4.2) [29].

## Results

### Screening and patient inclusion

**Fig. 1:**
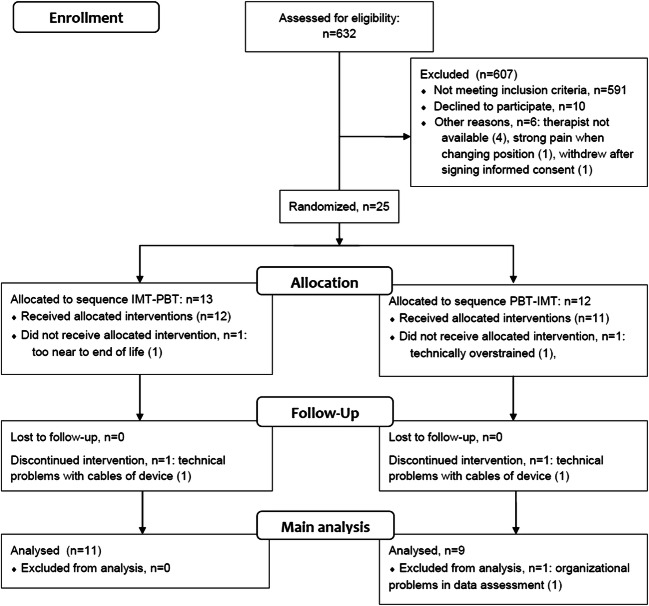
Flow diagram

Participants were recruited from February 2016 to February 2018. We screened 632 patients on the inpatient palliative care ward (see Fig. 1). Most of the patients (591/632, 93.5%) were not eligible (Fig. 1). Twenty-five of 41 (61.0%) eligible patients were randomized. Eleven patients in IMT-PBT and 9 in PBT-IMT completed the study (*N* = 20).

The dropout analysis is presented in detail in Online Resource 4. It shows that six of the 26 recruited patients (23.1%) were dropouts, one of them dropped out after signing informed consent but before randomization (see Fig. 1). These dropped out patients tended to have a higher Eastern Cooperative Oncology Group (ECOG) level and higher average pain levels before receiving PBT: 3.35 (standard deviation [SD] 1.35) vs. 5.25 (SD 0.5). The other variables were, for the most part, balanced.

Most patients (15/20, 75%) stopped the study after completing both sequences and were not available for the short-term follow-up, e.g., because of the burden in using TENS (5/15, 33%) or no perceived effect (3/15, 20%) (see Online Resource 5).

### Baseline characteristics

Table 1 shows the baseline characteristics in both sequences. Most characteristics were well balanced. Slight differences between both sequences were observed in cancer entity, ECOG, and DN4. Average pain at baseline was lower in the IMT-PBT than in the PBT-IMT sequence and, within the IMT-PBT sequence, lower in PBT than in IMT (see Table 1).

**Table 1:** Baseline characteristics

	Sequence IMT-PBT: *N* = 11	Sequence PBT-IMT: *N* = 9
Age, mean (SD)	58.3 (16.2)	59.2 (9.4)
Sex		
Male	4 (36.4%)	4 (44.4%)
Female	7 (63.6%)	5 (55.6%)
BMI, mean (SD)	23.6 (6.5)	23.5 (4.1)
ECOG		
1	0 (0.0%)	0 (0.0%)
2	7 (63.6%)	3 (33.3%)
3	4 (36.4%)	6 (66.7%)
4	0 (0.0%)	0 (0.0%)
Primary tumor		
Lung-Ca	3 (27.3%)	2 (22.2%)
Pancreas-Ca	2 (18.2%)	0 (0.0%)
Mamma-Ca	0 (0.0%)	1 (11.1%)
Prostate-Ca	0 (0.0%)	1 (11.1%)
Rectum-Ca	1 (9.1%)	0 (0.0%)
Miscellaneous	5 (45.5%)	5 (55.6%)
TENS position		
Lower limb	1 (9.1%)	0 (0.0%)
Lumbar spine	3 (27.3%)	2 (22.2%)
Pelvis	2 (18.2%)	2 (22.2%)
Ribs	4 (36.4%)	1 (11.1%)
Scapula	0 (0.0%)	1 (11.1%)
Thoracic spine	1 (9.1%)	3 (33.3%)
Radiation (not in TENS area)		
Yes	2 (18.2%)	2 (22.2%)
No	9 (81.8%)	7 (77.8%)
DN4 score		
DN4 < 4	8 (72.7%)	4 (44.4%)
DN4 ≥ 4	3 (27.3%)	5 (55.6%)
ECP mechanism of pain		
Nociceptive: visceral and/or bone or soft tissue	6 (54.5%)	4 (44.4%)
Neuropathic with or without nociceptive pain	5 (45.5%)	5 (55.6%)
ECP incident pain		
Yes	10 (90.9%)	7 (77.8%)
No	1 (9.1%)	2 (22.2%)
ECP psychological distress		
Yes	9 (81.8%)	7 (77.8%)
No	2 (18.2%)	1 (11.1%)
Insufficient information to classify	0 (0.0%)	1 (11.1%)
Non-physical effects on pain (“total pain”)		
No effect	1 (9.1%)	0 (0.0%)
Small effect	6 (54.5%)	4 (44.4%)
Moderate effect	3 (27.3%)	2 (22.2%)
Large effect	1 (9.1%)	3 (33.3%)
Large effect	0 (0.0%)	0 (0.0%)
Average pain before treatment IMT, mean (SD)	3.5 (1.0)	4.2 (0.8)
Average pain before treatment PBT, mean (SD)	2.6 (1.2)	4.2 (1.0)

*BMI*, body mass index; *DN4*, Douleur Neuropathique en 4 Questions; *ECOG*, Eastern Cooperative Oncology Group; *ECP*, Edmonton Classification System for Cancer Pain; *IMT*, intensity-modulated high TENS; *NRS*, numerical rating scale; *PBT*, placebo TENS; *SD*, standard deviation; *TENS*, transcutaneous electrical nerve stimulation

DN4 score: range 0–10: higher score = greater neuropathic pain (≥ 4 cutoff value for neuropathic pain)

ECOG: range 0–5. 0 = Fully active, able to carry on all pre-disease performance without restriction; 1 = restricted in physically strenuous activity but ambulatory and able to carry out work of a light or sedentary nature, e.g., light house work, office work; 2 = ambulatory and capable of all self-care but unable to carry out any work activities, up and about more than 50% of waking hours; 3 = capable of only limited self-care, confined to bed or chair more than 50% of waking hours; 4 = completely disabled, cannot carry on any self-care, totally confined to bed or chair; 5 = dead (Oken et al., 1982)

NRS for average pain: 0 = no pain or no interference; 10 = worst imaginable pain or maximum interference

Regular medication intake before randomization intervention was comparable between sequences (Online Resource 6). The oral MED per day had slightly higher means and SDs in the IMT-PBT sequence. Opioid, non-opioid, antidepressant, and anticonvulsant intake was equally distributed between IMT-PBT and PBT-IMT. None of these drugs were decreased by > 50% of the dose or removed during the trial. In IMT, increasing or adding drugs was not necessary. In PBT, some drugs had to be added or increased by > 50% of the dose: non-opioid 2/20 (10%), antidepressant 1/20 (5%), and anticonvulsant 1/20 (5%).

### Duration of TENS use

In sequence IMT-PBT, patients used TENS for 10.6 h (SD 8.3) in IMT and for 5.7 h (SD 5.0) in PBT based on the record from the TENS device. In contrast, the TENS use in sequence PBT-IMT was 7.1 h (SD 6.3) in IMT and 8.4 h (SD 6.2) in PBT. In total, TENS was used for 9.1 h (SD 7.5) in IMT and for 7.0 h (SD 5.6) in PBT during the 24-h period (*p* = 0.3340; *n* = 17; three missing values) (Online Resource 7).

### Difference *between* groups

Table 2 shows the change scores in periods, sequences, and the total for analyzing differences between the IMT and PBT phase in the primary and secondary outcomes.

**Table 2:** Differences between groups. Change scores in periods, sequences, and total (IMT-PBT: *N* = 11; PBT-IMT: *N* = 9; total: *N* = 20)

Outcome	Sequence	Period 1	Period 2	IMT minus PBT	Total IMT minus PBT	*p* value*
		Mean (SD)	Mean (SD)	Mean of differences (SD)	Mean of differences (95% CI)	
Average pain	IMT-PBT	− 0.8 (1.0)	− 0.4 (1.0)	− 0.5 (1.4)	− 0.2 (− 0.9 to 0.6)	0.6590
NRS: 0–10	PBT-IMT	− 1.2 (1.5)	− 1.0 (1.4)	0.2 (1.6)		
Worst pain	IMT-PBT	− 1.4 (1.7)	− 1.4 (2.1)	0.0 (2.6)	0.3 (− 1.1 to 1.6)	0.7125
NRS: 0–10	PBT-IMT	− 1.8 (3.1)	− 1.2 (1.3)	0.6 (3.6)		
Least pain	IMT-PBT	− 0.5 (1.1)	− 0.4 (1.2)	− 0.1 (2.0)	− 0.2 (− 1.1 to 0.7)	0.6295
NRS: 0–10	PBT-IMT	− 0.7 (1.4)	− 1.0 (0.9)	− 0.3 (1.7)		
Quality of life	IMT-PBT	0.7 (1.4)	0.1 (1.3)	0.6 (2.0)	− 0.1 (− 1.0 to 0.8)	0.8252
Scale: 1–7	PBT-IMT	0.9 (1.4)	− 0.1 (1.2)	− 1.0 (1.7)		
General activity	IMT-PBT	− 1.0 (3.2)	− 1.5 (2.6)	0.5 (4.5)	0.0 (− 2.2 to 2.2)	1.0000
NRS: 0–10	PBT-IMT	− 1.2 (4.0)	− 1.9 (2.9)	− 0.7 (5.1)		
Mood	IMT-PBT	0.0 (3.3)	− 0.5 (2.9)	0.5 (3.9)	0.3 (− 1.5 to 2.1)	0.7351
NRS: 0–10	PBT-IMT	− 2.1 (3.9)	− 2.0 (1.7)	0.1 (4.1)		
Walking ability	IMT-PBT	− 1.8 (3.9)	0.4 (2.1)	− 2.2 (4.5)	− 1.0 (− 2.9 to 1.0)	0.3229
NRS: 0–10	PBT-IMT	− 2.3 (3.7)	− 1.8 (2.2)	0.6 (3.5)		
Normal work	IMT-PBT	− 2.7 (3.6)	0.6 (2.9)	− 3.4 (5.5)	− 1.6 (− 4.0 to 0.8)	0.1745
NRS: 0–10	PBT-IMT	− 2.6 (3.1)	− 2.0 (2.1)	0.6 (3.8)		
Relations	IMT-PBT	0.7 (2.7)	0.1 (1.9)	0.6 (3.6)	− 0.4 (− 1.9 to 1.1)	0.5790
NRS: 0–10	PBT-IMT	0.0 (1.7)	− 1.7 (1.3)	− 1.7 (2.1)		
Sleep	IMT-PBT	− 1.0 (2.3)	0.7 (3.0)	− 1.7 (3.1)	− 0.8 (− 2.5 to 0.9)	0.3279
NRS: 0–10	PBT-IMT	− 1.7 (4.0)	− 1.3 (2.2)	0.3 (3.9)		
Enjoyment of life	IMT-PBT	− 0.1 (4.0)	0.0 (2.2)	− 0.1 (5.1)	− 0.1 (− 2.1 to 1.9)	0.9185
NRS: 0–10	PBT-IMT	− 1.7 (3.2)	− 1.8 (1.4)	− 0.1 (3.5)		

*EORTC QLQ-C30*, European Organization for Research and Treatment of Cancer quality-of-life questionnaire core 30; *IMT*, intensity-modulated high TENS; *NRS*, numerical rating scale; *PBT*, placebo TENS; *SD*, standard deviation; *TENS*, transcutaneous electrical nerve stimulation

NRS items adapted from the Brief Pain Inventory: 0 = no pain or no interference; 10 = worst imaginable pain or maximum interference; quality of life scale: 1 = very poor, 7 = excellent

Sequence IMT-PBT, *N* = 11; sequence PBT-IMT, *N* = 9; total, *N* = 20; quality of life was measured with the EORTC QLQ-C30 quality of life item; item “Pain relief with TENS” not listed: provides only post treatment values

*p value testing difference between treatments: paired *t* test of within-subject differences of pre-post change scores from both periods; *p* values < 0.05: statistically significant difference between treatments (Li et al., 2015); checking carry-over effect: unpaired *t* test of within-subject sums of the result from both periods: all *p* values were ≥ 0.05: carry-over effect is negligible (Wellek & Blettner, 2012)

The differences of IMT minus PBT for each sequence were rather small and none of them were statistically significant in this pilot trial. The results of the change scores were consistent with the sensitivity analysis of post treatment scores (Online Resource 8) [26].

**Fig. 2:**
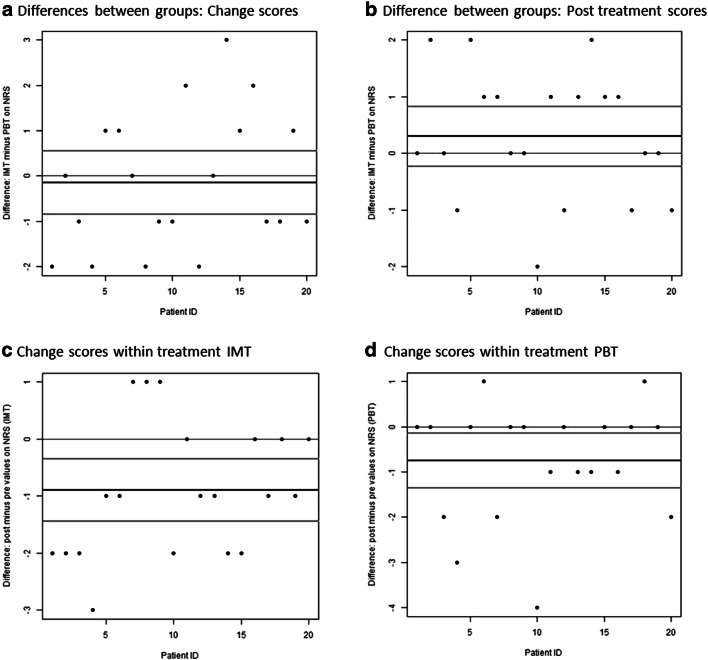
Graphical analysis of pain mean intensity. **a** Change scores (primary outcome). **b** Post treatment scores. **c** Change scores within IMT. **d** Change scores within PBT. IMT, intensity-modulated high TENS; NRS, numerical rating scale; PBT, placebo TENS. Negative values in **a** and **b** favor IMT; negative values in **c** favor IMT or in **d** PBT. Thin black line, null or no effect; dark gray line, mean of differences; light gray line, 95% confidence interval of paired *t* test (Li et al., 2015)

Figure 2a and b illustrates each participant’s change score and post treatment score, respectively, that were used to compare IMT and PBT (negative values favor IMT).

The change of pain perception during the TENS application is shown in Table 3. Seventeen out of 20 patients (85%) had at least a “slight improvement” on the 7-point VRS (responder criterion) during IMT and 10/20 (50%) during the PBT phase (*p* = 0.0428).

**Table 3:** Change on verbal rating scale (*N* = 20)

Category	IMT	PBT
Very clear deterioration	0 (0%)	1 (5%)
Clear deterioration	0 (0%)	0 (0%)
Slight deterioration	0 (0%)	0 (0%)
No change	2 (10%)	7 (35%)
Slight improvement	13 (65%)	9 (45%)
Clear improvement	3 (15%)	1 (5%)
Very clear improvement	1 (5%)	0 (0%)
Not applicable; no pain the last 24 h	1 (5%)	2 (10%)

*IMT*, intensity-modulated high TENS; *PBT*, placebo TENS

### Difference *within* groups

The analysis of changes within IMT indicated that patients experienced a decrease in average pain, worst pain, least pain, mood, walking ability, and relations (Table 4). In PBT, comparable changes were observed for average pain and worst pain but not for any of the other outcomes (Online Resource 9).

**Table 4:** Change scores within treatment IMT (*N* = 20)

Outcome	Pre mean (SD)	Post mean (SD)	Post–pre difference (95% CI)	*p* value*
Average pain NRS: 0–10	3.8 (1.0)	2.9 (1.2)	− 0.9 (− 1.4 to − 0.4)	0.0027
Worst pain NRS: 0–10	6.0 (1.7)	4.7 (2.2)	− 1.3 (− 2.0 to − 0.6)	0.0010
Least pain NRS: 0–10	2.2 (1.4)	1.5 (1.3)	− 0.7 (− 1.2 to − 0.2)	0.0068
Pain relief with TENS Scale: 0–100%	4.3 (1.5)	4.7 (0.9)	0.4 (− 0.3 to 1.0)	0.2601
Quality of life Scale: 0–7	4.9 (1.8)	3.5 (2.4)	− 1.4 (− 2.8 to 0.0)	0.0529
General activity NRS: 0–10	4.6 (2.2)	3.7 (2.6)	− 0.9 (− 2.2 to 0.4)	0.1707
Mood NRS: 0–10	4.5 (2.6)	2.7 (2.4)	− 1.8 (− 3.3 to − 0.3)	0.0206
Walking ability NRS: 0–10	5.7 (2.3)	3.3 (2.2)	− 2.4 (− 3.8 to − 1.0)	0.0017
Normal work NRS: 0–10	2.9 (2.3)	2.6 (2.3)	− 0.4 (− 1.5 to 0.8)	0.5314
Relations NRS: 0–10	3.3 (2.5)	2.1 (2.1)	− 1.2 (− 2.2 to − 0.1)	0.0310
Sleep NRS: 0–10	4.6 (2.7)	3.7 (3.0)	− 0.9 (− 2.3 to 0.6)	0.2399

*EORTC QLQ-C30*, European Organization for Research and Treatment of Cancer quality-of-life questionnaire core 30; *IMT*, intensity-modulated high TENS; *NRS*, numerical rating scale; *SD*, standard deviation; *TENS*, transcutaneous electrical nerve stimulation

NRS items adapted from the Brief Pain Inventory: 0 = no pain or no interference; 10 = worst imaginable pain or maximum interference. Pain relief: 0% = no pain relief; 100% = maximum pain relief. Quality of life scale: 1 = very poor, 7 = excellent

*p value of paired *t* test testing difference within treatment IMT

Figure 2 gives an overview of the change within groups for the primary outcome. Both IMT (Fig. 2c) and PBT (Fig. 2d) clearly included more negative change scores with slightly more decrease in average pain for IMT.

### Safety: TENS-related adverse events

One out of 20 (5%) patients perceived the electric current as uncomfortable after the IMT phase and 1/20 (5%) after the PBT phase. No other TENS-related adverse events were reported. Four patients (20%) generally criticized that cables were impractical and one (5%) patient felt disturbed by the electrodes.

After testing both TENS modes, 7/20 (35%) patients requested a prescription for the TENS device in order to use TENS after discharge.

### Explorative subgroup analysis

Online Resources 10, 11, 12, and 13 show the core outcomes for mean pain intensity (see Fig. 2), which were analyzed with regard to the patients’ benefit. Four patients in the change score comparison (Online Resource 10), one patient in the post treatment comparison (Online Resource 11), seven patients in the change within IMT comparison (Online Resource 12), and five patients in the change within PBT comparison (Online Resource 13) experienced a benefit, defined as difference or change of more than minus one on the NRS (see also Fig. 2). Taking the baseline values in Table 1 into account, descriptive comparisons indicated that females and patients suffering from incident pain were probably more likely to benefit from IMT although the sample sizes of these comparisons were very small (Online Resource 10, Online Resource 12). The explorative subgroup analysis did not indicate differences between patients with neuropathic and non-neuropathic pain.

## Discussion

### Summary of main findings

With regard to change in average pain intensity (primary outcome), we observed no differences between IMT and PBT. However, results indicated higher responder rates for IMT as secondary outcome. TENS was safe and well accepted.

### Difference between groups

There were no statistically significant differences between groups. These findings are supported by other RCTs, which evaluated TENS in cancer patients [9–11]. However, neither our study nor the other RCTs were powered to detect small effects between groups. In contrast, a recent, powered cross-over RCT with 40 head and neck cancer patients identified clinically relevant effects favoring active TENS compared with PBT and no TENS for resting pain [27, 30–32]. In this trial, the outcomes were assessed before and directly after 30 min of TENS. Change scores were compared among three TENS conditions (active TENS with 125 Hz, PBT, no TENS) and seven outcomes resulting in 21 statistical tests, which raises the question of a multiple testing problem in this analysis [33].

Interestingly, our results indicate that IMT had more responders than PBT as measured on the 7-point VRS (responder criterion: at least a “slight improvement”) for change of pain perception during TENS application. This important finding is strengthened by another cross-over RCT, in which the authors also observed a difference between active TENS and PBT on the VRS but not on the NRS scale [9]. Therefore, a 7-point VRS for pain relief might be more responsive than an 11-point NRS for assessing TENS in short period interventions [9].

### Difference within groups

In IMT, the items worst pain, mood, walking ability, and relations showed a mean change of one or more on an 11-point NRS in the IMT phase, which was suggested as clinically relevant [30]. Additionally, the mean changes of IMT in least pain, mood, walking ability, and relations may also be clinically relevant for individual patients, i.e., a change of two points or more on an 11-point NRS or a pain relief of 33% or more [27]. The change in walking ability for IMT underpins the idea that TENS may be helpful to reduce movement-related pain [9, 15].

For PBT, only changes in average pain and worst pain were observed. Similar to IMT, the changes in average pain were slightly below the threshold of clinical relevance as explained above [27, 30]. However, the change of − 1.6 (95% CI − 2.7 to − 0.4) in worst pain in the PBT phase can be considered clinically relevant [30].

Comparable changes within groups were also reported in other RCTs for average pain and worst pain [11], pain at rest [9, 31] or at movement [9], and fatigue [31].

The results underline that different outcome measures should be assessed since reduced pain perception could result in increased activity levels implying changes in physical function and psycho-social outcomes [10, 15].

### TENS-related adverse events

With regard to safety, IMT was well accepted and safe in this study. These results are comparable with the analysis of another RCT [9]. The six dropped out patients tended to have a higher ECOG level and higher average pain levels before receiving PBT. The other variables were balanced or hard to judge because of the low number of dropouts. The safety of TENS was not explicitly measured in some RCTs [10, 11, 31] but authors referred to TENS as safe method. A usability problem rather than a safety problem was the fact that the main reason for stopping the study after period 2 was the burden in using TENS (5/15, 33%), e.g., because of the disturbing cables of the device (see Online Resource 5 for further reasons).

### Gender aspects and incident pain

Among all benefit subgroup analyses, we believe that two findings are noteworthy even though the results were descriptive and the sample size was very small.

#### Gender

In palliative care and in the field of cancer pain management, gender issues are currently becoming increasingly recognized and future research in this area is advocated [34–36]. Interestingly, in our trial, women were more likely to report improvement from TENS. This could be a result of recently discussed sex differences regarding testosterone and estrogen levels as well as T cells and immune cells and their role in pain perception. However, most findings were based on animal models [37]. To the best of our knowledge, we are unaware of previous TENS RCTs in cancer pain reporting a gender differences in benefit subgroup analysis.

#### Incident pain

In the field of cancer pain, incident cancer pain has been identified as a condition with room for improvement in terms of the available treatment options [38]. Therefore, it should be noted that in our study, patients with incident cancer pain were more likely to experience benefit under TENS, which has not been assessed in previous TENS trials in palliative care [8]. A reason for this finding might be that demonstrating a change was easier in patients with increased pain scores than in the comparatively low baseline pain scores of patients without incident pain [9].

### Strengths

The strength of this study is the cross-over design that allowed patients to be their own control and have a balance of covariates [9]. The generalizability can be considered high because of the wide inclusion criteria, no artificial changes in patients’ medication plans, and the possibility of an individual use of TENS. The latter could also have had a positive impact on self-efficacy of the included cancer patients [39].

Though the washout phase of other RCTs [9–11, 31] differed from our trial, we are still confident that our study design and its washout phase was appropriate due to the absence of carry-over effects and washouts of 20–30 min that are considered adequate in literature [4, 40, 41].

Also, we considered methodological recommendations for clinical trials evaluating TENS for pain treatment that covered the domains allocation, application, and assessment [17]. Accordingly, sources of bias based on the Cochrane’s risk of bias tool for RCTs were thoroughly considered [42].

Finally, RCT data concerning TENS from the specialist palliative care setting is extremely scarce. Our study showed that using TENS was feasible in patients with advanced cancer on an inpatient palliative care ward. It provides valuable data on efficacy and safety, and hereby enhances the evidence base of TENS for advanced cancer patients.

### Limitations

A shortcoming of this study is that effects may have been underestimated because the pain intensity was low at baseline and outcomes were not measured during the peak effect of TENS, i.e., immediately after stopping TENS [15]. The responder definition in this trial was pre-specified in the protocol (NCT02655289) and based on the VRS. However, a responder definition based on the NRS was found more appropriate for patients’ chronic pain conditions [43].

Most patients had some sensation during the PBT phase and patients were told that two active TENS modes were compared. This may have led to the considerable effects within the PBT phase and, consequently, to small differences between groups. A third group, e.g., no TENS, could have been of help for assessing the possibly large placebo effect. In general, it should be considered that only a minority of the screened patients on the inpatient palliative care ward was eligible (41/632, 6.5%).

For the sake of generalizability, we included patients with various types of cancer, sites of pain and types of pain, which enhanced heterogeneity of the sample. In contrast to trials assessing pain therapy in chronic non-cancer pain conditions [44], the primary outcome in palliative cancer pain trials is probably more influenced by progress and instability of the disease that may have further contributed to the heterogeneity and variance in the results.

We closely followed the study protocol (NCT02655289). There were only two minor deviations: no block randomization and no recruitment via palliative care consultant service (see Online Resource 14).

We abstained from imputing missing data to enable an intention-to-treat-analysis because of the small sample and the pilot character of this trial [45]. Therefore, the results on efficacy and safety of TENS for advanced cancer pain patients, and especially the subgroup analyses, need to be interpreted cautiously and take into account that a type 2 error cannot be excluded.

## Conclusion

TENS was safe, but IMT was unlikely to offer more analgesic effects than PBT. As secondary outcomes, we found higher responder rates for IMT than for PBT and mean changes within both groups that may be clinically relevant for patients especially in the IMT group. Even though many patients desired to continue TENS therapy after the end of the study, 50% of the patients still reported at least “slight pain relief” from PBT. These results should be interpreted cautiously due to the per-protocol analysis and the small sample size of this trial, especially in the subgroup analyses. Nevertheless, we suggest that differences for gender and incident pain aspects should be further investigated in future trials.

## Electronic supplementary material

ESM 1 (DOCX 208 kb)

## Data Availability

The corresponding author has full control of all primary data. Primary data is available on request.
