# Supplementary material for: Transcutaneous electrical nerve stimulation for advanced cancer pain inpatients in specialist palliative care—a blinded, randomized, sham-controlled pilot cross-over trial
Source: Support Care Cancer. 2020 Mar 3;28(11):5323–33. doi: 10.1007/s00520-020-05370-8 (PMC7547037; doi:10.1007/s00520-020-05370-8)
Supplement: Supplementary file 1 — (DOCX 208 kb) [file 520_2020_5370_MOESM1_ESM.docx]

**Transcutaneous electrical nerve stimulation for advanced cancer pain inpatients in specialist palliative care – a blinded, randomized, sham-controlled pilot cross-over trial**

Waldemar Siemens,^1^ Christopher Boehlke,^1^ Michael I. Bennett,^2^ Klaus Offner,^3^ Gerhild Becker,^1^ Jan Gaertner^4^

^1^Clinic for Palliative Care, Medical Center, University of Freiburg, Faculty of Medicine, University of Freiburg, Freiburg, Germany

^2^Academic Unit of Palliative Care, Leeds Institute of Health Sciences (LIHS), School of Medicine, University of Leeds, Leeds, UK

^3^Department of Anesthesiology and Critical Care, Medical Center, University of Freiburg, Faculty of Medicine, University of Freiburg, Freiburg, Germany

^4^Center for Palliative Care Hildegard, Basel, Germany

**Corresponding author:** Waldemar Siemens

Clinic for Palliative Care, Medical Center, University of Freiburg,

Robert-Koch-Str 3, 79106 Freiburg, Germany

Phone: Tel: +49 761 270-95418, Fax: +49 761 270-94414

Email: waldemar.siemens@uniklinik-freiburg.de

ORCID iD: 0000-0003-4238-5327

**Journal:** Supportive Care in Cancer

**Content:**

[Online Resource 1: Consolidated Standards of Reporting Trials (CONSORT) Statement’s extension for non-pharmacological treatments (NPTs) 1](#_Toc27121620)

[Online Resource 2: The TIDieR (Template for Intervention Description and Replication) Checklist 6](#_Toc27121621)

[Online Resource 3: Study design 8](#_Toc27121622)

[Online Resource 4: Dropout: no versus yes 9](#_Toc27121623)

[Online Resource 5: Short-term follow-up 10](#_Toc27121624)

[Online Resource 6: Regular medication intake before randomization 11](#_Toc27121625)

[Online Resource 7: Self-reported vs. device-recorded time of TENS 12](#_Toc27121626)

[Online Resource 8: Difference between groups: Post treatment scores in periods, sequences and total (IMT-PBT: n=11; PBT-IMT: n=9; total: N=20) 13](#_Toc27121627)

[Online Resource 9: Benefit: Change scores within treatment PBT (N=20) 14](#_Toc27121628)

[Online Resource 10: Benefit: Change scores: Difference IMT minus PBT more than minus 1 15](#_Toc27121629)

[Online Resource 11: Benefit: Post treatment scores: Difference IMT minus PBT more than minus 1 16](#_Toc27121630)

[Online Resource 12: Benefit: Change within IMT more than minus 1 17](#_Toc27121631)

[Online Resource 13: Benefit: Change within PBT more than minus 1 18](#_Toc27121632)

[Online Resource 14: Differences between protocol and manuscript 19](#_Toc27121633)

# Online Resource 1: Consolidated Standards of Reporting Trials (CONSORT) Statement’s extension for non-pharmacological treatments (NPTs)

**2017 CONSORT checklist of information to include when reporting a randomized trial assessing nonpharmacologic treatments (NPTs)*. Modifications of the extension appear in italics and blue.**

| Section/Topic Item | Checklist item no. | CONSORT item | Extension for NPT trials | # page |
| --- | --- | --- | --- | --- |
| Title and abstract |  |  |  |  |
|  | 1a | Identification as a randomized trial in the title |  | 1 |
|  | 1b | Structured summary of trial design, methods, results, and conclusions (for specific guidance see CONSORT for abstracts) | *Refer to CONSORT extension for abstracts for NPT trials* | 2, 3 |
| Introduction |  |  |  |  |
| Background and objectives | 2a | Scientific background and explanation of rationale |  | 4 |
|  | 2b | Specific objectives or hypotheses |  | 4 |
| Methods |  |  |  |  |
| Trial design | 3a | Description of trial design (such as parallel, factorial) including allocation ratio | When applicable, how care providers were allocated to each trial group | 4, 5 |
|  | 3b | Important changes to methods after trial commencement (such as eligibility criteria), with reasons |  | n. a. |
| Participants | 4a | Eligibility criteria for participants | When applicable, eligibility criteria for centers and for *care providers* | 5 |
|  | 4b | Settings and locations where the data were collected |  | 4, 7, 8 |
| Interventions*†* | 5 | The interventions for each group with sufficient details to allow replication, including how and when they were actually administered | Precise details of both the experimental treatment and comparator | 6, Online Resource 2 |
|  | 5a |  | Description of the different components of the interventions and, when applicable, description of the procedure for tailoring the interventions to individual participants. | 6, Online Resource 2 |
|  | 5b |  | Details *of whether and* how the interventions were standardized. | 6, Online Resource 2 |
|  | 5c. |  | Details *of whether and* how adherence of care providers to the protocol was assessed or enhanced | 7, 8, Online Resource 2 |
|  | 5d |  | *Details of whether and how adherence of participants to interventions was assessed or enhanced* | 7, 8 |
| Outcomes | 6a | Completely defined pre-specified primary and secondary outcome measures, including how and when they were assessed |  | 6, 7 |
|  | 6b | Any changes to trial outcomes after the trial commenced, with reasons |  | n. a. |
| Sample size | 7a | How sample size was determined | When applicable, details of whether and how the clustering by care providers or centers was addressed | 8 |
|  | 7b | When applicable, explanation of any interim analyses and stopping guidelines |  | n. a. |
| Randomization: |  |  |  |  |
| - Sequence generation | 8a | Method used to generate the random allocation sequence |  | 7, 8 |
|  | 8b | Type of randomization; details of any restriction (such as blocking and block size) |  | 7, 8 |
| - Allocation concealment mechanism | 9 | Mechanism used to implement the random allocation sequence (such as sequentially numbered containers), describing any steps taken to conceal the sequence until interventions were assigned |  | 7, 8 |
| - Implementation | 10 | Who generated the random allocation sequence, who enrolled participants, and who assigned participants to interventions |  | 7, 8 |
| Blinding | 11a | If done, who was blinded after assignment to interventions (for example, participants, care providers, those assessing outcomes) and how | If done, who was blinded after assignment to interventions (e.g., participants, care providers, *those administering co-interventions,* those assessing outcomes) and how | 7, 8 |
|  | 11b | If relevant, description of the similarity of interventions |  | 6-8 |
|  | 11c |  | *If blinding was not possible, description of any attempts to limit bias* | n. a. |
| Statistical methods | 12a | Statistical methods used to compare groups for primary and secondary outcomes | When applicable, details of whether and how the clustering by care providers or centers was addressed | 8, 9 |
|  | 12b | Methods for additional analyses, such as subgroup analyses and adjusted analyses |  | 8, 9 |
| Results |  |  |  |  |
| Participant flow (a diagram is strongly recommended) | 13a | For each group, the numbers of participants who were randomly assigned, received intended treatment, and were analyzed for the primary outcome | The number of care providers or centers performing the intervention in each group and the number of patients treated by each care provider or in each center | 9, 10, Figure 2 |
|  | 13b | For each group, losses and exclusions after randomization, together with reasons |  | 9, 10, Figure 2, Online Resource 3 |
|  | 13c |  | *For each group, the delay between randomization and the initiation of the intervention* | 7, 8 |
|  | new |  | Details of the experimental treatment and comparator as they were implemented | 10, Table 1 |
| Recruitment | 14a | Dates defining the periods of recruitment and follow-up |  | 9 |
|  | 14b | Why the trial ended or was stopped |  | n.a. |
| Baseline data | 15 | A table showing baseline demographic and clinical characteristics for each group | When applicable, a description of care providers (case volume, qualification, expertise, etc.) and centers (volume) in each group. | 10, Table 1 |
| Numbers analyzed | 16 | For each group, number of participants (denominator) included in each analysis and whether the analysis was by original assigned groups |  | 9, 10, Figure 2 |
| Outcomes and estimation | 17a | For each primary and secondary outcome, results for each group, and the estimated effect size and its precision (such as 95% confidence interval) |  | 11, 12, Table 3, Table 4, Table 5, Table 6 |
|  | 17b | For binary outcomes, presentation of both absolute and relative effect sizes is recommended |  | n. a. |
| Ancillary analyses | 18 | Results of any other analyses performed, including subgroup analyses and adjusted analyses, distinguishing pre-specified from exploratory |  | 12 |
| Harms | 19 | All important harms or unintended effects in each group (for specific guidance see CONSORT for harms) |  | 12 |
| **Discussion** |  |  |  |  |
| Limitations | 20 | Trial limitations, addressing sources of potential bias, imprecision, and, if relevant, multiplicity of analyses | In addition, take into account the choice of the comparator, lack of or partial blinding, and unequal expertise of care providers or centers in each group | 16 |
| Generalizability | 21 | Generalizability (external validity, applicability) of the trial findings | Generalizability (external validity) of the trial findings according to the intervention, comparators, patients, and care providers and centers involved in the trial | 15, 16 |
| Interpretation | 22 | Interpretation consistent with results, balancing benefits and harms, and considering other relevant evidence |  | 12-15 |
| Other information |  |  |  |  |
| Registration | 23 | Registration number and name of trial registry |  | 4 |
| Protocol | 24 | Where the full trial protocol can be accessed, if available |  | 4, NCT02655289 |
| Funding | 25 | Sources of funding and other support (such as supply of drugs), role of funders |  | 19 |

*Additions or modifications to the 2010 CONSORT checklist. CONSORT = Consolidated Standards of Reporting Trials

†The items 5, 5a, 5b, 5c, 5d are consistent with the Template for Intervention Description and Replication (TIDieR) checklist

n. a.: not applicable

# Online Resource 2: The TIDieR (Template for Intervention Description and Replication) Checklist

**
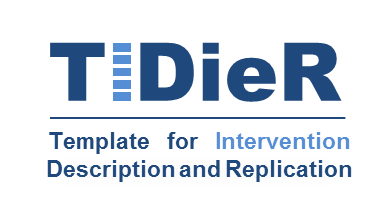
The TIDieR (Template for Intervention Description and Replication) Checklist*:**

Information to include when describing an intervention and the location of the information

| **Item number** | **Item** | **Where located **** | |
| --- | --- | --- | --- |
|  |  | Primary paper  (page) | Other ^†^ (details) |
|  | **BRIEF NAME** |  |  |
| **1.** | Provide the name or a phrase that describes the intervention. | 6 | - |
|  | **WHY** |  |  |
| **2.** | Describe any rationale, theory, or goal of the elements essential to the intervention. | 4, 6 | - |
|  | **WHAT** |  |  |
| **3.** | Materials: Describe any physical or informational materials used in the intervention, including those provided to participants or used in intervention delivery or in training of intervention providers. Provide information on where the materials can be accessed (e.g. online appendix, URL). | 6 | - |
| **4.** | Procedures: Describe each of the procedures, activities, and/or processes used in the intervention, including any enabling or support activities. | 7, 8 | - |
|  | **WHO PROVIDED** |  |  |
| **5.** | For each category of intervention provider (e.g. psychologist, nursing assistant), describe their expertise, background and any specific training given. | 7, 8 | intervention provided by an experienced sport scientist |
|  | **HOW** |  |  |
| **6.** | Describe the modes of delivery (e.g. face-to-face or by some other mechanism, such as internet or telephone) of the intervention and whether it was provided individually or in a group. | 6-8 | - |
|  | **WHERE** |  |  |
| **7.** | Describe the type(s) of location(s) where the intervention occurred, including any necessary infrastructure or relevant features. | 4 | - |
|  | **WHEN and HOW MUCH** |  |  |
| **8.** | Describe the number of times the intervention was delivered and over what period of time including the number of sessions, their schedule, and their duration, intensity or dose. | 6 | - |
|  | **TAILORING** |  |  |
| **9.** | If the intervention was planned to be personalised, titrated or adapted, then describe what, why, when, and how. | 6 | - |
|  | **MODIFICATIONS** |  |  |
| **10.^ǂ^** | If the intervention was modified during the course of the study, describe the changes (what, why, when, and how). | n. a. | - |
|  | **HOW WELL** |  |  |
| **11.** | Planned: If intervention adherence or fidelity was assessed, describe how and by whom, and if any strategies were used to maintain or improve fidelity, describe them. | n. a. (individual use of TENS device) | The self-reported and device-recorded time was documented |
| **12.^ǂ^** | Actual: If intervention adherence or fidelity was assessed, describe the extent to which the intervention was delivered as planned. | n. a. (individual use of TENS device) |  |

** **Authors** - use N/A if an item is not applicable for the intervention being described. **Reviewers** – use ‘?’ if information about the element is not reported/not sufficiently reported.

† If the information is not provided in the primary paper, give details of where this information is available. This may include locations such as a published protocol or other published papers (provide citation details) or a website (provide the URL).

ǂ If completing the TIDieR checklist for a protocol, these items are not relevant to the protocol and cannot be described until the study is complete.

* We strongly recommend using this checklist in conjunction with the TIDieR guide (see *BMJ* 2014;348:g1687) which contains an explanation and elaboration for each item.

* The focus of TIDieR is on reporting details of the intervention elements (and where relevant, comparison elements) of a study. Other elements and methodological features of studies are covered by other reporting statements and checklists and have not been duplicated as part of the TIDieR checklist. When a **randomised trial** is being reported, the TIDieR checklist should be used in conjunction with the CONSORT statement (see [www.consort-statement.org](http://www.consort-statement.org)) as an extension of **Item 5 of the CONSORT 2010 Statement.** When a **clinical trial** **protocol** is being reported, the TIDieR checklist should be used in conjunction with the SPIRIT statement as an extension of **Item 11 of the SPIRIT 2013 Statement** (see [www.spirit-statement.org](http://www.spirit-statement.org)). For alternate study designs, TIDieR can be used in conjunction with the appropriate checklist for that study design (see [www.equator-network.org](http://www.equator-network.org)).

n. a.: not applicable

# Online Resource 3: Study design


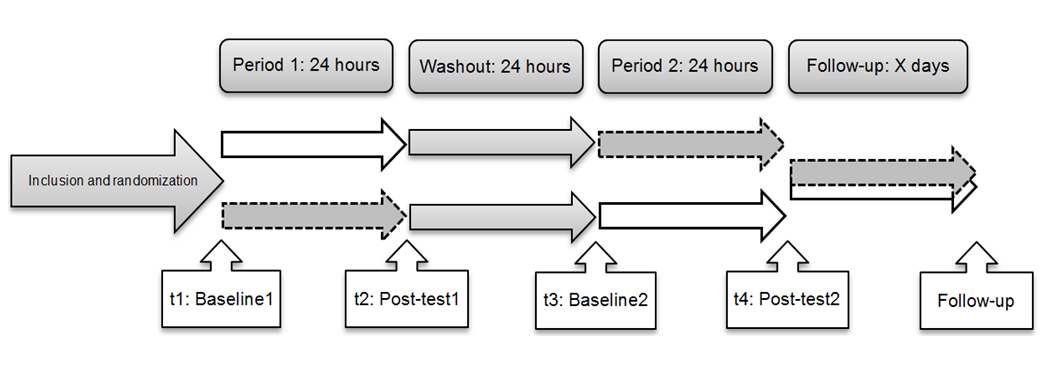


# Online Resource 4: Dropout: no versus yes

|  | **no N=20** | **yes N=6** |
| --- | --- | --- |
| Age, mean (SD) | 58.7 (13.2) | 59.8 (7.57) |
| Sex |  |  |
| male | 8 (40.0%) | 2 (33.3%) |
| female | 12 (60.0%) | 4 (66.7%) |
| BMI, mean (SD) | 23.5 (5.42) | 27.0 (7.7) |
| ECOG: |  |  |
| 1 | 0 (0.0%) | 0 (0.0%) |
| 2 | 10 (50.0%) | 2 (33.3%) |
| 3 | 10 (50.0%) | 1 (16.7%) |
| 4 | 0 (0.0%) | 3 (50.0%) |
| Primary tumor |  |  |
| lung-Ca | 5 (25.0%) | 0 (0.0%) |
| pancreas-Ca | 2 (10.0%) | 0 (0.0%) |
| mamma-Ca | 1 (5.0%) | 3 (50.0%) |
| prostate-Ca | 1 (5.0%) | 0 (0.0%) |
| rectum-Ca | 1 (5.0%) | 2 (33.3%) |
| miscellaneous | 10 (50.0%) | 1 (16.7%) |
| TENS position: |  |  |
| lower limb | 1 (5.00%) | 1 (16.7%) |
| lumbar spine | 5 (25.0%) | 1 (16.7%) |
| pelvis | 4 (20.0%) | 0 (0.0%) |
| ribs | 5 (25.0%) | 0 (0.0%) |
| scapula | 1 (5.0%) | 0 (0.0%) |
| thoracic spine | 4 (20.0%) | 0 (0.0%) |
| shoulder | 0 (0.0%) | 1 (16.7%) |
| lateral upper brachium | 0 (0.0%) | 2 (33.3%) |
| Dropout before decision for TENS-position | 0 (0.0%) | 1 (16.7%) |
| Radiation (not in TENS area): |  |  |
| yes | 4 (20.0%) | 3 (50.0%) |
| no | 16 (80.0%) | 3 (50.0%) |
| DN4 Score: |  |  |
| DN4 < 4 | 12 (60.0%) | 4 (66.7%) |
| DN4 ≥ 4 | 8 (40.0%) | 2 (33.3%) |
| ECP mechanism of pain: |  |  |
| nociceptive: visceral and/or bone or soft tissue | 10 (50.0%) | 4 (66.7%) |
| neuropathic with or without nociceptive pain | 10 (50.0%) | 2 (33.3%) |
| ECP incident pain: |  |  |
| yes | 17 (85.0%) | 4 (66.7%) |
| no | 3 (15.0%) | 2 (33.3%) |
| ECP psychological distress: |  |  |
| yes | 16 (80.0%) | 5 (83.3%) |
| no | 3 (15.0%) | 1 (16.7%) |
| insufficient information to classify | 1 (5.0%) | 0 (0.0%) |
| Non-physical effects on pain (“total pain”): |  |  |
| no effect | 1 (5.0%) | 0 (0.0%) |
| small effect | 10 (50.0%) | 3 (50.0%) |
| moderate effect | 5 (25.0%) | 1 (16.7%) |
| large effect | 4 (20.0%) | 1 (16.7%) |
| very large effect | 0 (0.0%) | 1 (16.7%) |
| Average pain before treatment IMT, mean (SD) | 3.80 (1.01) | 3.67 (1.53) |
| Average pain before treatment PBT, mean (SD) | 3.35 (1.35) | 5.25 (0.50) |

BMI: body mass index; DN4: Douleur Neuropathique en 4 Questions; ECOG: Eastern Cooperative Oncology Group; ECP: Edmonton Classification System for Cancer Pain; IMT: intensity-modulated high TENS; NRS: numerical rating scale; PBT: placebo TENS; SD: standard deviation; TENS: transcutaneous electrical nerve stimulation

DN4 Score: range 0-10: higher score = greater neuropathic pain (≥4 cut-off value for neuropathic pain)

ECOG: range 0-5: 0 = Fully active, able to carry on all pre-disease performance without restriction; 1 = Restricted in physically strenuous activity but ambulatory and able to carry out work of a light or sedentary nature, e.g., light house work, office work; 2 = Ambulatory and capable of all self-care but unable to carry out any work activities, up and about more than 50% of waking hours; 3 = Capable of only limited self-care, confined to bed or chair more than 50% of waking hours; 4 = Completely disabled; cannot carry on any selfcare; totally confined to bed or chair; 5 = Dead (Oken et al., 1982)

NRS for average pain: 0 = no pain or no interference; 10 = worst imaginable pain or maximum interference

# Online Resource 5: Short-term follow-up

**Reason for or against follow-up: Sequence IMT-PBT**

| **Patient** | **Follow-up** | **Reason for or against follow-up: Sequence IMT-PBT (n=11)** |
| --- | --- | --- |
| 1 | no | No reason stated |
| 2 | yes | Hope that there will be relief |
| 3 | yes | Thinks longer-term application could be better |
| 4 | yes | Pain reduction; safe freedom of movement |
| 5 | no | No improvement during strong pain; the device distracts from the actual pain when pain sensation is low |
| 6 | yes | No reason stated |
| 7 | no | Stimulation current is disturbing when I want to rest; One cannot know if TENS is the reason for the effect; A lot of effort to apply TENS; would be easier to get a detailed user guide from the study (no flexibility) |
| 8 | no | No reason stated |
| 9 | no | No reason stated |
| 10 | no | Is not beneficial |
| 11 | no | Too many cables or tubes of medication; Potential to be at home with a prescription to try out |

IMT: intensity-modulated high TENS; PBT: placebo TENS

**Reason for or against follow-up: Sequence PBT-IMT**

| **Patient** | **Follow-up** | **Reason for or against follow-up: Sequence PBT-IMT (n=9)** |
| --- | --- | --- |
| 1 | no | Pain seems well-adjusted |
| 2 | no | Corset system planned; Note: rating difficult because of shortness of breath on Friday |
| 3 | no | “Test completely without”* |
| 4 | no | Does not get along with the device so well alone |
| 5 | no | Movement is restricted by the cables |
| 6 | yes | Pain relief in arms and legs |
| 7 | no | End of stay; no use at home because cable are disturbing |
| 8 | no | Wants to use TENS on other parts of the body |
| 9 | no | No effect |

* Statement unclear

IMT: intensity-modulated high TENS; PBT: placebo TENS

# Online Resource 6: Regular medication intake before randomization

| **Medication** | **Sequence IMT-PBT: N=11** | **Sequence PBT-IMT: N=9** |
| --- | --- | --- |
| Morphine equivalent in mg/day: IMT, mean (SD) | 226.8 (222.6) | 205.7 (132.1) |
| Morphine equivalent in mg/day: PBT, mean (SD) | 230.3 (215.7) | 209.6 (143.3) |
| Opioids: | 10 (100%) | 9 (100%) |
| yes | 10 (100%) | 9 (100%) |
| no | 0 (0.0%) | 0 (0.0%) |
| Non-opioids |  |  |
| yes | 8 (80.0%) | 8 (88.9%) |
| no | 2 (20.0%) | 1 (11.1%) |
| Antidepressants |  |  |
| yes | 5 (50.0%) | 4 (44.4%) |
| no | 5 (50.0%) | 5 (55.6%) |
| Anticonvulsants: |  |  |
| yes | 6 (60.0%) | 6 (66.7%) |
| no | 4 (40.0%) | 3 (33.3%) |

IMT: intensity-modulated high; SD: standard deviation; PBT: placebo TENS

# Online Resource 7: Self-reported vs. device-recorded time of TENS

| **Group** | **Self-reported time of TENS use** | **Device-recorded time of TENS use** | **p value** |
| --- | --- | --- | --- |
| **IMT** | 10.5 (SD 6.7) | 8.8 (SD 7.6) | 0.0610 |
|  | mean difference: 1.71 (95% CI: 0.09 to 3.50) | |  |
| **PBO** | 5.2 (SD 3.8) | 7.4 (SD 5.7) | 0.2847 |
|  | mean difference: -2.24 (95% CI: -6.52 to 2.05) | |  |
| **Total** | 7.9 (SD 6.0) | 8.1 (SD 6.7) | 0.8165 |
|  | mean difference: -0.27 (95% CI: -2.57 to 2.04) | |  |

CI: confidence interval; IMT: intensity-modulated high TENS; PBT: placebo TENS; SD: standard deviation

Results based on unpaired t-test of 34 complete-cases for self-reported and device-recorded time of TENS use (6 missing values)

# Online Resource 8: Difference between groups: Post treatment scores in periods, sequences and total (IMT-PBT: n=11; PBT-IMT: n=9; total: N=20)

| **Outcome** | **Sequence** | **Period 1:**  **mean (SD)** | **Period 2**  **mean (SD)** | **IMT minus PBT**  **mean of differences (SD)** | **Total IMT minus PBT**  **mean of differences (95% CI)** | **p value*** |  |
| --- | --- | --- | --- | --- | --- | --- | --- |
| Average pain | IMT-PBT | 2.6 (0.9) | 2.3 (1.3) | 0.4 (1.2) | 0.3 (-0.2 to 0.8) | 0.2492 |  |
| NRS: 0-10 | PBT-IMT | 3.0 (1.5) | 3.2 (1.5) | 0.2 (1.1) |  |  |  |
| Worst pain | IMT-PBT | 4.1 (1.3) | 3.1 (1.6) | 1.0 (1.6) | 0.7 (-0.6 to 2.0) | 0.2731 |  |
| NRS: 0-10 | PBT-IMT | 5.0 (2.5) | 5.3 (3.0) | 0.3 (3.8) |  |  |  |
| Least pain | IMT-PBT | 1.6 (1.4) | 1.0 (1.2) | 0.6 (1.3) | 0.1 (-0.6 to 0.8) | 0.7547 |  |
| NRS: 0-10 | PBT-IMT | 1.9 (1.4) | 1.3 (1.2) | -0.6 (1.3) |  |  |  |
| Quality of life | IMT-PBT | 4.6 (0.9) | 4.5 (1.2) | 0.1 (1.0) | -0.1 (-0.7 to 0.5) | 0.7157 |  |
| Scale: 1-7 | PBT-IMT | 5.0 (0.7) | 4.7 (1.0) | -0.3 (1.4) |  |  |  |
| General activity | IMT-PBT | 4.0 (2.5) | 2.1 (1.8) | 1.9 (2.5) | 0.8 (-0.7 to 2.2) | 0.2993 |  |
| NRS: 0-10 | PBT-IMT | 3.6 (2.6) | 2.9 (2.2) | -0.7 (3.4) |  |  |  |
| Mood | IMT-PBT | 4.6 (2.8) | 3.0 (2.4) | 1.6 (2.7) | 0.8 (-0.6 to 2.1) | 0.2674 |  |
| NRS: 0-10 | PBT-IMT | 2.8 (2.6) | 2.4 (1.7) | -0.3 (3.0) |  |  |  |
| Walking ability | IMT-PBT | 3.6 (2.7) | 3.7 (3.1) | -0.1 (3.8) | -0.6 (-2.0 to 0.9) | 0.4276 |  |
| NRS: 0-10 | PBT-IMT | 2.6 (2.2) | 1.4 (1.4) | -1.1 (1.8) |  |  |  |
| Normal work | IMT-PBT | 3.9 (2.5) | 3.7 (3.3) | 0.2 (3.7) | -0.4 (-1.9 to 1.1) | 0.5904 |  |
| NRS: 0-10 | PBT-IMT | 3.6 (2.6) | 2.4 (1.7) | -1.1 (2.8) |  |  |  |
| Relations | IMT-PBT | 3.4 (2.2) | 2.3 (2.5) | 1.1 (3.3) | 0.3 (-1.1 to 1.6) | 0.6987 |  |
| NRS: 0-10 | PBT-IMT | 2.3 (2.4) | 1.6 (2.0) | -0.8 (1.9) |  |  |  |
| Sleep | IMT-PBT | 2.3 (2.3) | 2.6 (3.0) | -0.4 (3.7) | -0.7 (-2.2 to 0.9) | 0.3774 |  |
| NRS: 0-10 | PBT-IMT | 2.9 (2.3) | 1.9 (2.0) | -1.0 (2.7) |  |  |  |
| Enjoyment of life | IMT-PBT | 5.3 (2.7) | 2.5 (2.5) | 2.8 (3.4) | 1.2 (-0.4 to 2.8) | 0.1404 |  |
| NRS: 0-10 | PBT-IMT | 2.6 (2.7) | 1.8 (2.0) | -0.8 (2.5) |  |  |  |
| Pain relief with TENS | IMT-PBT | 35.5 (26.2) | 15.5 (16.9) | 20.0 (26.1) | 8.0 (-7.3 to 23.3) | 0.2876 | |
| NRS: 0-100% | PBT-IMT | 34.4 (31.7) | 27.8 (18.6) | -6.7 (35.4) |  |  |  |

EORTC QLQ-C30: European Organisation for Research and Treatment of Cancer Quality-of-life Questionnaire Core 30; IMT: intensity-modulated high TENS; NRS: numerical rating scale; PBT: placebo TENS; SD: standard deviation; TENS: transcutaneous electrical nerve stimulation

11-point-NRS Items adapted from the Brief Pain Inventory: 0 = no pain or no interference; 10 = worst imaginable pain or maximum interference; Pain relief: 11-point-NRS: 0% = no pain relief; 100% = maximum pain relief; quality of life scale: 1 = very poor, 7 = excellent

Sequence IMT-PBT: N=11; sequence PBT-IMT: N=9; Total: N 20; Quality of life was measured with the EORTC QLQ-C30 quality of life item

*p value testing difference between treatments: paired t-test of within-subject differences of post treatment scores from both periods, p values <0.05: statistically significant difference between treatments (Li et al, 2015); checking carry-over effect: unpaired t-test of within-subject sums of the result from both periods: all p values were ≥0.05: carry-over effect is negligible (Wellek & Blettner, 2012)

# Online Resource 9: Benefit: Change scores within treatment PBT (N=20)

| **Outcome** | **Pre mean (SD)** | **Post mean (SD)** | **Post – pre difference (95% CI)** | **p value** |
| --- | --- | --- | --- | --- |
| Average pain  NRS: 0-10 | 3.4 (1.3) | 2.6 (1.4) | -0.8 (-1.4 to -0.1) | 0.0178 |
| Worst pain  NRS: 0-10 | 5.5 (2.3) | 4.0 (2.3) | -1.6 (-2.7 to -0.4) | 0.0122 |
| Least pain  NRS: 0-10 | 1.9 (1.2) | 1.4 (1.3) | -0.5 (-1.1 to 0.1) | 0.0961 |
| Pain relief with TENS  Scale: 0-100% | 4.3 (1.3) | 4.8 (1.0) | 0.5 (-0.2 to 1.1) | 0.1543 |
| Quality of life  Scale: 0-7 | 4.2 (2.7) | 2.8 (2.2) | -1.4 (-2.9 to 0.1) | 0.0641 |
| General activity  NRS: 0-10 | 4.1 (3.0) | 2.9 (2.4) | -1.2 (-2.8 to 0.4) | 0.1290 |
| Mood  NRS: 0-10 | 4.1 (3.4) | 3.2 (2.8) | -0.9 (-2.3 to 0.6) | 0.2399 |
| Walking ability  NRS: 0-10 | 4.5 (3.2) | 3.7 (2.9) | -0.8 (-2.4 to 0.8) | 0.2967 |
| Normal work  NRS: 0-10 | 2.3 (2.2) | 2.3 (2.4) | 0.1 (-0.8 to 0.9) | 0.9003 |
| Relations  NRS: 0-10 | 3.1 (3.1) | 2.8 (2.6) | -0.4 (-2.0 to 1.3) | 0.6688 |
| Sleep  NRS: 0-10 | 3.3 (2.6) | 2.5 (2.5) | -0.8 (-2.0 to 0.5) | 0.2345 |

CI: confidence interval; EORTC QLQ-C30: European Organisation for Research and Treatment of Cancer Quality-of-life Questionnaire Core 30; NRS: numerical rating scale; PBT: placebo TENS; SD: standard deviation; TENS: transcutaneous electrical nerve stimulation

NRS Items adapted from the Brief Pain Inventory: 0 = no pain or no interference; 10 = worst imaginable pain or maximum interference; Pain relief: 0% = no pain relief; 100% = maximum pain relief; quality of life scale: 1 = very poor, 7 = excellent

* p value of paired t-test testing difference within treatment PBT

# Online Resource 10: Benefit: Change scores: Difference IMT minus PBT more than minus 1

|  | **no N=16** | **yes N=4** |
| --- | --- | --- |
| Sequence: |  |  |
| IMT-PBT | 10 (62.5%) | 1 (25.0%) |
| PBT-IMT | 6 (37.5%) | 3 (75.0%) |
| Age, mean (SD) | 58.6 (14.2) | 59.2 (10.0) |
| Sex |  |  |
| male | 7 (43.8%) | 1 (25.0%) |
| female | 9 (56.2%) | 3 (75.0%) |
| BMI, mean (SD) | 23.5 (5.8) | 23.5 (4.2) |
| ECOG: |  |  |
| 1 | 0 (0.0%) | 0 (0.0%) |
| 2 | 8 (50.0%) | 2 (50.0%) |
| 3 | 8 (50.0%) | 2 (50.0%) |
| 4 | 0 (0.0%) | 0 (0.0%) |
| Primary tumor |  |  |
| lung-Ca | 4 (25.0%) | 1 (25.0%) |
| pancreas-Ca | 2 (12.5%) | 0 (0.0%) |
| mamma-Ca | 0 (0.0%) | 1 (25.0%) |
| prostate-Ca | 0 (0.0%) | 1 (25.0%) |
| rectum-Ca | 1 (6.3%) | 0 (0.0%) |
| miscellaneous | 9 (56.2%) | 1 (25.0%) |
| TENS position |  |  |
| lower limb | 1 (6.3%) | 0 (0.0%) |
| lumbar spine | 5 (31.2%) | 0 (0.0%) |
| pelvis | 4 (25.0%) | 0 (0.0%) |
| ribs | 4 (25.0%) | 1 (25.0%) |
| scapula | 0 (0.0%) | 1 (25.0%) |
| thoracic spine | 2 (12.5%) | 2 (50.0%) |
| Radiation (not in TENS area): |  |  |
| yes | 3 (18.8%) | 1 (25.0%) |
| no | 13 (81.2%) | 3 (75.0%) |
| DN4 Score: |  |  |
| DN4 < 4 | 10 (62.5%) | 2 (50.0%) |
| DN4 ≥ 4 | 6 (37.5%) | 2 (50.0%) |
| ECP mechanism of Pain: |  |  |
| nociceptive: visceral and/or bone or soft tissue | 8 (50.0%) | 2 (50.0%) |
| neuropathic with or without nociceptive pain | 8 (50.0%) | 2 (50.0%) |
| ECP incident Pain: |  |  |
| yes | 13 (81.2%) | 4 (100%) |
| no | 3 (18.8%) | 0 (0.00%) |
| ECP psychological distress: |  |  |
| yes | 13 (81.2%) | 3 (75.0%) |
| no | 3 (18.8%) | 0 (0.0%) |
| insufficient information to classify | 0 (0.00%) | 1 (25.0%) |
| Non-physical effects on pain (Total pain): |  |  |
| no effect | 1 (6.3%) | 0 (0.0%) |
| small effect | 7 (43.8%) | 3 (75.0%) |
| moderate effect | 4 (25.0%) | 1 (25.0%) |
| large effect | 4 (25.0%) | 0 (0.0%) |
| Average pain before treatment IMT, mean (SD) | 3.75 (1.00) | 4.00 (1.15) |
| Average pain before treatment PBT, mean (SD) | 3.38 (1.31) | 3.25 (1.71) |
| Morphine equivalent in mg/day: IMT, mean (SD) | 215.0 (202.6) | 226.8 (84.4) |
| Morphine equivalent in mg/day: PBT, mean (SD) | 226.2 (202.7) | 200.2 (65.2) |

BMI: body mass index; DN4: Douleur Neuropathique en 4 Questions; ECOG: Eastern Cooperative Oncology Group; ECP: Edmonton Classification System for Cancer Pain; IMT: intensity-modulated high TENS; NRS: numerical rating scale; PBT: placebo TENS; SD: standard deviation

DN4 Score: range 0-10: higher score = greater neuropathic pain (≥4 cut-off value for neuropathic pain)

ECOG: range 0-5: 0 = Fully active, able to carry on all pre-disease performance without restriction; 1 = Restricted in physically strenuous activity but ambulatory and able to carry out work of a light or sedentary nature, e.g., light house work, office work; 2 = Ambulatory and capable of all self-care but unable to carry out any work activities, up and about more than 50% of waking hours; 3 = Capable of only limited self-care, confined to bed or chair more than 50% of waking hours; 4 = Completely disabled; cannot carry on any selfcare; totally confined to bed or chair; 5 = Dead (Oken et al., 1982)

NRS for average pain: 0 = no pain or no interference; 10 = worst imaginable pain or maximum interference

# Online Resource 11: Benefit: Post treatment scores: Difference IMT minus PBT more than minus 1

|  | **no N=19** | **yes N=1** |
| --- | --- | --- |
| Sequence: |  |  |
| IMT-PBT | 11 (57.9%) | 0 (0.0%) |
| PBT-IMT | 8 (42.1%) | 1 (100%) |
| Age, mean (SD) | 59.2 (13.4) | 50.0 (.) |
| Sex |  |  |
| male | 8 (42.1%) | 0 (0.0%) |
| female | 11 (57.9%) | 1 (100%) |
| BMI, mean (SD) | 23.2 (5.3) | 30.1 (.) |
| ECOG: |  |  |
| 1 | 0 (0.0%) | 0 (0.0%) |
| 2 | 10 (52.6%) | 0 (0.0%) |
| 3 | 9 (47.4%) | 1 (100%) |
| 4 | 0 (0.0%) | 0 (0.0%) |
| Primary tumor |  |  |
| lung-Ca | 4 (21.1%) | 1 (100%) |
| pancreas-Ca | 2 (10.5%) | 0 (0.0%) |
| mamma-Ca | 1 (5.3%) | 0 (0.0%) |
| prostate-Ca | 1 (5.3%) | 0 (0.0%) |
| rectum-Ca | 1 (5.3%) | 0 (0.0%) |
| miscellaneous | 10 (52.6%) | 0 (0.0%) |
| TENS position |  |  |
| lower limb | 1 (5.3%) | 0 (0.0%) |
| lumbar spine | 4 (21.1%) | 1 (100%) |
| pelvis | 4 (21.1%) | 0 (0.0%) |
| ribs | 5 (26.3%) | 0 (0.0%) |
| scapula | 1 (5.3%) | 0 (0.0%) |
| thoracic spine | 4 (21.1%) | 0 (0.0%) |
| Radiation (not in TENS area): |  |  |
| yes | 4 (21.1%) | 0 (0.0%) |
| no | 15 (78.9%) | 1 (100%) |
| DN4 Score: |  |  |
| DN4 < 4 | 12 (63.2%) | 0 (0.0%) |
| DN4 ≥ 4 | 7 (36.8%) | 1 (100%) |
| ECP mechanism of Pain: |  |  |
| nociceptive: visceral and/or bone or soft tissue | 10 (52.6%) | 0 (0.0%) |
| neuropathic with or without nociceptive pain | 9 (47.4%) | 1 (100%) |
| ECP incident Pain: |  |  |
| yes | 16 (84.2%) | 1 (100%) |
| no | 3 (15.8%) | 0 (0.0%) |
| ECP psychological distress: |  |  |
| yes | 15 (78.9%) | 1 (100%) |
| no | 3 (15.8%) | 0 (0.0%) |
| insufficient information to classify | 1 (5.3%) | 0 (0.0%) |
| Non-physical effects on pain (Total pain): |  |  |
| no effect | 1 (5.3%) | 0 (0.0%) |
| small effect | 10 (52.6%) | 0 (0.0%) |
| moderate effect | 5 (26.3%) | 0 (0.0%) |
| large effect | 3 (15.8%) | 1 (100%) |
| Average pain before treatment IMT, mean (SD) | 3.79 (1.03) | 4.00 (.) |
| Average pain before treatment PBT, mean (SD) | 3.26 (1.33) | 5.00 (.) |
| Morphine equivalent in mg/day: IMT, mean (SD) | 212.1 (186.6) | 317.0 (.) |
| Morphine equivalent in mg/day: PBT, mean (SD) | 209.4 (179.6) | 441.0 (.) |

BMI: body mass index; DN4: Douleur Neuropathique en 4 Questions; ECOG: Eastern Cooperative Oncology Group; ECP: Edmonton Classification System for Cancer Pain; IMT: intensity-modulated high TENS; NRS: numerical rating scale; PBT: placebo TENS; SD: standard deviation

DN4 Score: range 0-10: higher score = greater neuropathic pain (≥4 cut-off value for neuropathic pain)

ECOG: range 0-5: 0 = Fully active, able to carry on all pre-disease performance without restriction; 1 = Restricted in physically strenuous activity but ambulatory and able to carry out work of a light or sedentary nature, e.g., light house work, office work; 2 = Ambulatory and capable of all self-care but unable to carry out any work activities, up and about more than 50% of waking hours; 3 = Capable of only limited self-care, confined to bed or chair more than 50% of waking hours; 4 = Completely disabled; cannot carry on any selfcare; totally confined to bed or chair; 5 = Dead (Oken et al., 1982)

NRS for average pain: 0 = no pain or no interference; 10 = worst imaginable pain or maximum interference

# Online Resource 12: Benefit: Change within IMT more than minus 1

|  | **no N=13** | **yes N=7** |
| --- | --- | --- |
| Sequence: |  |  |
| IMT-PBT | 8 (61.5%) | 3 (42.9%) |
| PBT-IMT | 5 (38.5%) | 4 (57.1%) |
| Age, mean (SD) | 57.5 (13.6) | 60.9 (13.4) |
| Sex |  |  |
| male | 6 (46.2%) | 2 (28.6%) |
| female | 7 (53.8%) | 5 (71.4%) |
| BMI, mean (SD) | 23.9 (6.1) | 22.8 (4.1) |
| ECOG: |  |  |
| 1 | 0 (0.0%) | 0 (0.0%) |
| 2 | 7 (53.8%) | 3 (42.9%) |
| 3 | 6 (46.2%) | 4 (57.1%) |
| 4 | 0 (0.0%) | 0 (0.0%) |
| Primary tumor |  |  |
| lung-Ca | 2 (15.4%) | 3 (42.9%) |
| pancreas-Ca | 1 (7.7%) | 1 (14.3%) |
| mamma-Ca | 0 (0.00%) | 1 (14.3%) |
| prostate-Ca | 1 (7.7%) | 0 (0.0%) |
| rectum-Ca | 1 (7.7%) | 0 (0.0%) |
| miscellaneous | 8 (61.5%) | 2 (28.6%) |
| TENS position |  |  |
| lower limb | 1 (7.7%) | 0 (0.0%) |
| lumbar spine | 3 (23.1%) | 2 (28.6%) |
| pelvis | 3 (23.1%) | 1 (14.3%) |
| ribs | 3 (23.1%) | 2 (28.6%) |
| scapula | 1 (7.7%) | 0 (0.0%) |
| thoracic spine | 2 (15.4%) | 2 (28.6%) |
| Radiation (not in TENS area): |  |  |
| yes | 4 (30.8%) | 0 (0.0%) |
| no | 9 (69.2%) | 7 (100%) |
| DN4 Score: |  |  |
| DN4 < 4 | 9 (69.2%) | 3 (42.9%) |
| DN4 ≥ 4 | 4 (30.8%) | 4 (57.1%) |
| ECP mechanism of Pain: |  |  |
| nociceptive: visceral and/or bone or soft tissue | 8 (61.5%) | 2 (28.6%) |
| neuropathic with or without nociceptive pain | 5 (38.5%) | 5 (71.4%) |
| ECP incident Pain: |  |  |
| yes | 10 (76.9%) | 7 (100%) |
| no | 3 (23.1%) | 0 (0.0%) |
| ECP psychological distress: |  |  |
| yes | 11 (84.6%) | 5 (71.4%) |
| no | 1 (7.7%) | 2 (28.6%) |
| insufficient information to classify | 1 (7.7%) | 0 (0.00%) |
| Non-physical effects on pain (Total pain): |  |  |
| no effect | 1 (7.7%) | 0 (0.0%) |
| small effect | 5 (38.5%) | 5 (71.4%) |
| moderate effect | 4 (30.8%) | 1 (14.3%) |
| large effect | 3 (23.1%) | 1 (14.3%) |
| Average pain before treatment IMT, mean (SD) | 3.54 (0.88) | 4.29 (1.11) |
| Average pain before treatment PBT, mean (SD) | 3.46 (1.27) | 3.14 (1.57) |
| Morphine equivalent in mg/day: IMT, mean (SD) | 223.0 (220.0) | 206.8 (96.2) |
| Morphine equivalent in mg/day: PBT, mean (SD) | 227.3 (214.3) | 209.2 (114.7) |

BMI: body mass index; DN4: Douleur Neuropathique en 4 Questions; ECOG: Eastern Cooperative Oncology Group; ECP: Edmonton Classification System for Cancer Pain; IMT: intensity-modulated high TENS; NRS: numerical rating scale; PBT: placebo TENS; SD: standard deviation

DN4 Score: range 0-10: higher score = greater neuropathic pain (≥4 cut-off value for neuropathic pain)

ECOG: range 0-5: 0 = Fully active, able to carry on all pre-disease performance without restriction; 1 = Restricted in physically strenuous activity but ambulatory and able to carry out work of a light or sedentary nature, e.g., light house work, office work; 2 = Ambulatory and capable of all self-care but unable to carry out any work activities, up and about more than 50% of waking hours; 3 = Capable of only limited self-care, confined to bed or chair more than 50% of waking hours; 4 = Completely disabled; cannot carry on any selfcare; totally confined to bed or chair; 5 = Dead (Oken et al., 1982)

NRS for average pain: 0 = no pain or no interference; 10 = worst imaginable pain or maximum interference

# Online Resource 13: Benefit: Change within PBT more than minus 1

|  | **no N=15** | **yes N=5** |
| --- | --- | --- |
| Sequence: |  |  |
| IMT-PBT | 9 (60.0%) | 2 (40.0%) |
| PBT-IMT | 6 (40.0%) | 3 (60.0%) |
| Age, mean (SD) | 57.3 (13.2) | 62.8 (14.1) |
| Sex |  |  |
| male | 7 (46.7%) | 1 (20.0%) |
| female | 8 (53.3%) | 4 (80.0%) |
| BMI, mean (SD) | 22.5 (4.4) | 26.7 (7.4) |
| ECOG: |  |  |
| 1 | 0 (0.0%) | 0 (0.0%) |
| 2 | 9 (60.0%) | 1 (20.0%) |
| 3 | 6 (40.0%) | 4 (80.0%) |
| 4 | 0 (0.0%) | 0 (0.0%) |
| Primary tumor |  |  |
| lung-Ca | 3 (20.0%) | 2 (40.0%) |
| pancreas-Ca | 2 (13.3%) | 0 (0.00%) |
| mamma-Ca | 0 (0.0%) | 1 (20.0%) |
| prostate-Ca | 1 (6.7%) | 0 (0.0%) |
| rectum-Ca | 1 (6.7%) | 0 (0.0%) |
| miscellaneous | 8 (53.3%) | 2 (40.0%) |
| TENS position |  |  |
| lower limb | 1 (6.7%) | 0 (0.0%) |
| lumbar spine | 3 (20.0%) | 2 (40.0%) |
| pelvis | 2 (13.3%) | 2 (40.0%) |
| ribs | 5 (33.3%) | 0 (0.0%) |
| scapula | 1 (6.7%) | 0 (0.0%) |
| thoracic spine | 3 (20.0%) | 1 (20.0%) |
| Radiation (not in TENS area): |  |  |
| yes | 3 (20.0%) | 1 (20.0%) |
| no | 12 (80.0%) | 4 (80.0%) |
| DN4 Score: |  |  |
| DN4 < 4 | 8 (53.3%) | 4 (80.0%) |
| DN4 ≥ 4 | 7 (46.7%) | 1 (20.0%) |
| ECP mechanism of Pain: |  |  |
| nociceptive: visceral and/or bone or soft tissue | 10 (66.7%) | 0 (0.00%) |
| neuropathic with or without nociceptive pain | 5 (33.3%) | 5 (100%) |
| ECP incident Pain: |  |  |
| yes | 13 (86.7%) | 4 (80.0%) |
| no | 2 (13.3%) | 1 (20.0%) |
| ECP psychological distress: |  |  |
| yes | 12 (80.0%) | 4 (80.0%) |
| no | 2 (13.3%) | 1 (20.0%) |
| insufficient information to classify | 1 (6.7%) | 0 (0.00%) |
| Non-physical effects on pain (Total pain): |  |  |
| no effect | 1 (6.7%) | 0 (0.0%) |
| small effect | 7 (46.7%) | 3 (60.0%) |
| moderate effect | 4 (26.7%) | 1 (20.0%) |
| large effect | 3 (20.0%) | 1 (20.0%) |
| Average pain before treatment IMT, mean (SD) | 3.67 (0.9) | 4.20 (1.30) |
| Average pain before treatment PBT, mean (SD) | 3.07 (1.44) | 4.20 (0.45) |
| Morphine equivalent in mg/day: IMT, mean (SD) | 225.7 (198.0) | 192.1 (145.2) |
| Morphine equivalent in mg/day: PBT, mean (SD) | 226.8 (193.5) | 203.6 (162.1) |

BMI: body mass index; DN4: Douleur Neuropathique en 4 Questions; ECOG: Eastern Cooperative Oncology Group; ECP: Edmonton Classification System for Cancer Pain; IMT: intensity-modulated high TENS; NRS: numerical rating scale; PBT: placebo TENS; SD: standard deviation

DN4 Score: range 0-10: higher score = greater neuropathic pain (≥4 cut-off value for neuropathic pain)

ECOG: range 0-5: 0 = Fully active, able to carry on all pre-disease performance without restriction; 1 = Restricted in physically strenuous activity but ambulatory and able to carry out work of a light or sedentary nature, e.g., light house work, office work; 2 = Ambulatory and capable of all self-care but unable to carry out any work activities, up and about more than 50% of waking hours; 3 = Capable of only limited self-care, confined to bed or chair more than 50% of waking hours; 4 = Completely disabled; cannot carry on any selfcare; totally confined to bed or chair; 5 = Dead (Oken et al., 1982)

NRS for average pain: 0 = no pain or no interference; 10 = worst imaginable pain or maximum interference

# [Online Resource 14: Differences between protocol and manuscript](#_Toc3800263)

| **Protocol** | **Difference in publication** |
| --- | --- |
| Patients should have been recruited on the acute palliative care ward, the palliative care consultant service and the acute pain service. | We decided not to recruit patients from the palliative care consultant service due to organizational reasons. |
| The strata for randomization should have been pain on NRS of 3 to 4 and NRS ≥5. | The randomization process was applied without using the strata. |
